# Supplementary material for: The utility of MEWS for predicting the mortality in the elderly adults with COVID-19: a retrospective cohort study with comparison to other predictive clinical scores
Source: PeerJ. 2020 Sep 28;8:e10018. doi: 10.7717/peerj.10018 (PMC7528814; doi:10.7717/peerj.10018)
Supplement: Supplemental Information 1 — APACHE II score, CURB-65 score, PSI score, SOFA score, MEWS score, qSOFA score and SIRS Criteria. [file peerj-08-10018-s001.pdf]

Diagnosis and clinical classification of COVID-19-According to the Chinese Clinical Guidance for COVID-19 Pneumonia Diagnosis and Treatment 5th edition

I. Case Definitions

Outside the epicenter of Hubei province:

1. Suspect cases

Considering both the following epidemiological history and clinical manifestations:

1.1 Epidemiological history

1.1.1 History of travel to or residence in Wuhan and its surrounding areas, or in other communities where cases have been reported within 14 days prior to the onset of the disease;

1.1.2 In contact with novel coronavirus infected people (with positive results for the nucleic acid test) within 14 days prior to the onset of the disease;

1.1.3 In contact with patients who have fever or respiratory symptoms from Wuhan and its surrounding area, or from communities where confirmed cases have been reported within 14 days before the onset of the disease;

1.1.4 Clustered cases

1.2 Clinical manifestations

1.2.1 Fever and/or respiratory symptoms;

1.2.2 The aforementioned imaging characteristics of NCP;

1.2.3 Normal or decreased WBC count, normal or decreased lymphocyte count in the early stage of onset.

A suspect case has any of the epidemiological history plus any two clinical manifestations or all

three clinical manifestations if there is no clear epidemiological history.

2. Confirmed cases

Suspect cases with one of the following etiological or serological evidences:

2.1 Real-time fluorescent RT-PCR indicates positive for new coronavirus nucleic acid;

2.2 Viral gene sequence is highly homologous to known new coronaviruses.

Hubei province

1.Suspect cases

Considering both the following epidemiological history and clinical manifestations:

1.1 Epidemiological history

1.1.1 History of travel to or residence in Wuhan and its surrounding areas, or in other communities where cases have been reported within 14 days prior to the onset of the disease;

1.1.2 In contact with novel coronavirus infected people (with positive results for the nucleic acid test) within 14 days prior to the onset of the disease;

1.1.3 In contact with patients who have fever or respiratory symptoms from Wuhan and its surrounding area, or from communities where confirmed cases have been reported within 14 days before the onset of the disease;

1.1.4 Clustered cases

1.2 Clinical manifestations

1.2.1 Fever and/or respiratory symptoms;

1.2.2 Normal or decreased WBC count, normal or decreased lymphocyte count in the early stage of onset.

With or without any of the epidemiological history plus both of the clinical manifestations.

2.Clinically confirmed cases

Suspect cases with the aforementioned imaging characteristics of NCP;

3.Confirmed cases

Suspect cases or clinically confirmed cases with one of the following etiological or serological evidences:

3.1 Real-time fluorescent RT-PCR indicates positive for new coronavirus nucleic acid;

3.2 Viral gene sequence is highly homologous to known new coronaviruses.

Ⅱ. Clinical Classification

1.Mild cases

The clinical symptoms were mild, and there was no sign of pneumonia on imaging.

2.Moderate cases

Showing fever and respiratory symptoms with radiological findings of pneumonia.

3.Severe cases

Adult cases meeting any of the following criteria:

(1) Respiratory distress (≧30 breaths/ min);

(2) Oxygen saturation≤93% at rest;

(3) Arterial partial pressure of oxygen (PaO2)/ fraction of inspired oxygen (FiO2)≦ 300mmHg (l mmHg=0.133kPa).

4. Critical cases

Cases meeting any of the following criteria:

4.1 Respiratory failure and requiring mechanical ventilation;

4.2 Shock;

4.3 With other organ failure that requires ICU care.

Supplementary Text S2 APACHE II score, CURB-65 score, PSI score, SOFA score, MEWS score, qSOFA score, and SIRS Criteria

Text S2.1 Acute Physiology and Chronic Health Evaluation Ⅱ (APACHE Ⅱ)

• Use the worst value for each physiological variable during the first 24 hours after admission to ICU.

**Physiological variable**	**+4**	**+3**	**+2**	**+1**	**+0**	**+1**	**+2**	**+3**	**+4**
Temperature – rectal °C	≥41	39–40.9		38.5–38.9	36–38.4	34–35.9	32–33.9	30–31.9	≤29.9
Mean arterial pressure – mmHg	≥160	130–159	110–129		70–109		50–69		≤49
Heart rate (ventricular response)	≥180	140–179	110–139		70–109		55–69	40–54	≤39
Respiratory rate (non-ventilated or ventilated)	≥50	35–49		25–34	12–24	10–11	6–9		≤5
Oxygenation A. aDO_2 or PaO_2 (mmHg) FiO_2 ≥0.5 record A. aDO_2	≥500	350–499	200–349		< 200				
FiO_2 < 0.5 record only					PO_2 > 70	PO_2 61–		PO_2 55–60	PO_2 < 55

PaO_2						70			
Arterial pH	≥7.7	7.6–7.69		7.5–7.59	7.33–7.49		7.25–7.32	7.15–7.24	< 7.15
Serum sodium (mmol/L)	≥180	160–179	155–159	150–154	130–149		120–129	111–119	≤110
Serum potassium (mmol/L)	≥7	6–6.9		5.5–5.9	3.5–5.4	3–3.4	2.5–2.9		< 2.5
Serum creatinine (mg/100mL) (Double point score for acute renal failure)	≥3.5	2–3.4	1.5–1.9		0.6–1.4		< 0.6		
Hematocrit %	≥60		50–59.9	46–49.9	30–45.9		20–29.9		< 20
White blood count (total/mm^3)	≥40		20–39.9	15–19.9	3–14.9		1–2.9		< 1
Glasgow coma score (GCS)	Glasgow coma score (GCS) Score=15 minus actual GCS								
Serum HCO_2 (venous mmol/L) (Not	≥52	41–51.9		32–40.9	23–31.9		18–21.9	15–17.9	< 15

preferred, use if no ABGs)									

Acute Physiology and Chronic Health Evaluation Ⅱ: Previous Health Status				
**B Age Points**		**C Chronic Health Points**		**Score**
Assign points to age as follows		If the patient has a history of severe organ system insufficiency or is immune-compromised assign points as follows<br>a: for nonoperative or emergency postoperative patients – 5 points **or**<br>b: for elective postoperative patients – 2 points<br>**Definitions**<br>Organ insufficiency or immunocompromised state must have been evident prior to this hospital admission and conform to the following criteria:<br>Liver – biopsy proven cirrhosis and documented portal hypertension; episodes of past upper GI bleeding attributed to portal hypertension; or prior	Respiratory–Chronic restrictive, obstructive, or vascular disease resulting in severe exercise restriction (i.e., unable to climb stairs or perform household duties); or documented chronic hypoxia, hypercapnia, secondary polycythemia, severe pulmonary hypertension (> 40 mmHg), or respirator dependency.<br>Renal – receiving chronic dialysis. Immunocompromised – the patient has received therapy that suppresses resistance to infection (e.g., immunosuppression, chemotherapy, radiation, long term or recent high dose steroids, or has a disease that is sufficiently advanced to	Sum of A+B+C
Age (yrs)	Points			A APS points
≤44	0			B Age points
45–54	2			C Chronic Health points
55–64	3			
65–74	5			Total APACHE Ⅱ
≥75	6			

		episodes of hepatic failure/encephalopathy/coma Cardiovascular – New York Heart Association Class IV.	suppress resistance to infection, e.g., leukemia, lymphoma, AIDS).	

Glasgow coma score (GCS)

Factors	points					
	1	2	3	4	5	6
Eye Opening Response	No eye opening	To pain	To verbal command	Spontaneously	——	——
Verbal Response	No verbal response	Incomprehensible sounds	Inappropriate words	Confused	Oriented	
Motor Response	No motor response	Extension to pain	Flexion to pain	Withdraw from pain	Localizes pain	Obeys commands

Text S2.2 CURB-65 score

Factors	Points	
Confusion	1	
BUN > 19 mg/dL (> 7 mmol/L)	1	
Respiratory Rate ≥ 30	1	
Systolic BP < 90 mmHg or Diastolic BP ≤ 60 mmHg	1	
Age ≥ 65	1	
Total score		
CURB-65 Score	mortality Risk, (%)	Recommendation
0	0.9%	Low risk, Outpatient care
1	5.2%	Low risk, Inpatient vs. observation admission
2	12%	
3 or 4	31.2%	Inpatient admission with consideration for ICU admission with score of 4 or 5
The CURB-65 Score includes points for confusion and blood urea nitrogen, respiratory rates, blood pressure, and age.		

Text S2.3 Pneumonia Severity Index (PSI)

**Factor**	**PSI score**
Patient age	Age in years (male) or age −10 (female)
Nursing home resident	+10
**Coexisting illnesses**	
Neoplastic disease	+30
Liver disease	+20
Congestive cardiac failure	+10
Cerebrovascular disease	+10
Renal disease	+10
**Signs on examination**	
Altered mental state	+20
Respiratory rate ≥30 per minute	+20
Systolic blood pressure < 90 mmHg	+20
Temperature ≤35°C or ≥40°C	+15
Pulse rate ≥125 bpm	+10
**Results of investigations**	
Arterial pH < 7.35	+30
Serum urea level ≥11 mmol/l	+20
Serum sodium level < 130 mmol/	+10
Serum glucose level ≥14 mmol/lL	+10
Hematocrit < 30%	+10
PO_2 < 60 mmHg or O_2 saturation < 90%	+10
Pleural effusion	+10

Text S2.4 SOFA Score

**Variables**	**0**	**1**	**2**	**3**	**4**
Respiration					
PaO_2/FiO_2 (mmHg)	> 400	≤400	≤300	≤200 (with respiratory support)	≤100
Coagulation					
Platelets × 10^3/ml	> 150	≤150	≤100	≤50	≤20
Liver					
Bilirubin mg/dl mol/liter	< 1.2	1.2–1.9	2.0–5.9	6.0–11.9	> 12.0
	< 20	20–32	33–101	102–204	> 204
Cardiovascular					
Hypotension	No hypotension	MAP < 70 mmHg	Dopamine ≤5 dobutamine (any dose) [a]	Dopamine > 5 or epinephrine≤0.1 or norepinephrine ≤0.1[a]	Dopamine > 15 or epinephrine > 0.1 or norepinephrine > 0.1[a]
Central nervous system					
Glasgow Coma Score	15	13–14	10–12	6–9	< 6
Renal					
Cr mg/dl	< 1.2	1.2–1.9	2.0–3.4	3.5–4.9	≥5.0

mol/liter	< 110	110–170	171–299	300–440	> 440
Urine output	–	–	–	< 500 ml/day	< 200 ml/day

a: Adrenergic agents administered for at least 1 hr (doses given are in µg/kg/min).

Text S2.5 Modified Early Warning Score (MEWS score)

**Score**	**3**	**2**	**1**	**0**	**1**	**2**	**3**
Respiratory rate (min^{-1})		≤ 8		9–14	15–20	21–29	> 29
Heart rate (min^{-1})		≤ 40	41–50	51–100	101–110	111–129	> 129
Systolic BP (mmHg)	≤ 70	71–80	81–100	101–199		≥ 200	
Urine output (ml/kg/h)	Nil	< 0.5					
Temperature (°C)		≤ 35	35.1–36	36.1–38	38.1–38.5	≥ 38.6	
Neurological				Alert	Reacting to voice	Reacting to pain	Unresponsive

The scores for each parameter are recorded at the time that observations are taken. If the total is 4 or more then the ward doctor is informed.

Text S2.6 SIRS Criteria (≥ 2 meets SIRS definition)

Temperature	≤ 36° C or ≥ 38° C
Heart Rate	≥ 90 bpm
Respiratory Rate	≥ 20 breaths/min **or** $PaCO_2$ < 32 mmHg
White Blood Cell Count	≥ 12,000 or ≤ 4,000 cells/mm^3 **or** > 10% bands

Text S2.7 qSOFA (Quick SOFA) Score

Altered mental status GCS<15	No	Yes
Respiratory rate ≥22	No	Yes
Systolic BP ≤100	No	Yes

A “positive” qSOFA Score (≥2) suggests high risk of poor outcome in patients with suspected infection.
