# Supplementary material for: The utility of MEWS for predicting the mortality in the elderly adults with COVID-19: a retrospective cohort study with comparison to other predictive clinical scores
Source: PeerJ. 2020 Sep 28;8:e10018. doi: 10.7717/peerj.10018 (PMC7528814; doi:10.7717/peerj.10018)
Supplement: Supplemental Information 2 — NPV, negative predictive value; PPV, positive predictive value [file peerj-08-10018-s002.docx]

**Table A.1 ROC of MEWS in COVID-19 Patients between Male Patients Age≥75 Years and the Others.**

|  | **AUROC (95% CI)** | **Cut-off** | **Mortality ＞cut-off** | **Mortality ＜ cut-off** | **SEN (%)** | **SPE (%)** | **NPV (%)** | **PPV (%)** |
| --- | --- | --- | --- | --- | --- | --- | --- | --- |
| Male Patients Age≥75 | .92 (.86, .98) | 2.5 | 57.9% | 0.0% | 84.6 | 84.3 | 97.2 | 45.8 |
| Others | .91 (.83, 1.00) | 3.5 | 45.8% | 2.4% | 100.0 | 75.0 | 100.0 | 57.9 |

NPV: Negative predictive value; PPV: Positive predictive value.
